# Supplementary material for: A Formative Study of the Implementation of Whole Genome Sequencing in Northern Ireland
Source: Genes (Basel). 2022 Jun 21;13(7):1104. doi: 10.3390/genes13071104 (PMC9316942; doi:10.3390/genes13071104)
Supplement: Supplementary file 1 [file genes-13-01104-s001.zip › genes-1751660-supplementary.pdf]

Table S1: Participant survey demographics summary

**Participant demographic**	**Count**	**Percentage (%)**
Participant sex		
- Male	6	18.8
- Female	25	78.1
- Unknown	1	3.1
Survey completed by parent / carer	18	56.3
Age bracket of person unable to complete the form themselves		
- 0 – 17	13	72.2
- 18+	5	27.8
Age bracket of participant		
- 18-34	11	34.4
- 35-44	5	15.6
- 45-54	7	21.9
- 55+	8	25.0
- Skipped question	1	3.1
Ethnicity		
- British	8	25.0
- Northern Irish	18	56.3
- Other	5	15.6
- Skipped question	1	3.1
Medical specialism(s) attended		
- *Cardiology*	4	13.8
- *Dermatology*	5	17.2
- *Ear, nose and throat*	5	17.2
- *Gastroenterology*	5	17.2
- *Genetics*	18	62.1
- *General practitioner*	22	75.9
- *Haematology*	1	3.4
- *Immunology*	3	10.3
- *Nephrology/Urology*	4	13.8
- *Neurology*	14	48.3
- *Oncology*	2	6.9
- *Ophthalmology*	4	13.8
- *Paediatrics*	11	37.9
- *Psychiatry*	4	13.8
- *Respiratory*	5	17.2
- *Rheumatology*	1	3.4
- *Other*	7	24.1

Table S2: Themes and associated codes from semi-structured interviews of healthcare professionals

**Theme**	**Associated codes**
Healthcare professionals had a largely positive experience of the 100KGP	- Felt the project ran smoothly / enjoyed participation<br>- Discussed the significant benefits for the patients *e.g.* ending the diagnostic odyssey / modifying care plans<br>- Reported that they felt the participants also had a good experience of the 100KGP<br>- Emphasised willingness to participate in future similar projects<br>- The multi-disciplinary approach was useful<br>- Felt that WGS was the future of healthcare
Facilitating WGS was a significant workload burden	- Time-consuming<br>- Involved extra hours<br>- Difficult to manage on top of regular workload<br>- Capacity was limited
Interviewees found that participants expressed some concerns about additional findings and time to results	- Concerns about additional findings<br>- Concerns about time to results / management of expectations<br>- Generally good understanding<br>- Consent process could be streamlined / excess paperwork
There is a need for additional training	- Assumptions were made about genetics understanding beyond specialists<br>- Complicated field to understand<br>- Mainstreaming beyond genetics is needed<br>- Needs to be more straight-forward<br>- Training for non-specialists needed<br>- Concerns patients attending non-specialists may be at a disadvantage<br>- Flow chart of next steps may be useful

Table S3: Themes and associated codes from discussion workshop

**Themes**	**Codes**
Theme 1: Resource constraints hinder collaborative rare disease research	• Lack of collaboration<br>• Lack of funding<br>• Lack of political will to provide resources<br>• Lack of incentive for clinical research<br>• Cross border relationships in research enable better utilisation of limited resources on whole island<br>• Incentives needed to encourage research, both as a career and within clinical environments
Theme 2: Collaborative rare disease research is hindered by ineffective communication	• Lack of progress<br>• Lack of collaboration<br>• Lack of follow up and dissemination of results<br>• Multidisciplinary approach is needed<br>• Importance of communicating with patients effectively<br>• Need to establish better communication procedures to maintain contact between patients and healthcare professionals<br>• Wider support services need to be available to aid patients/families in processing a diagnosis

Theme 3: Rare disease awareness, support and information services are insufficient.	• Lack of community rare disease awareness / outreach<br>• Need for more communication and engagement on social media<br>• More comprehensive support services need to be available to aid patients/families in processing a diagnosis<br>• Need for more awareness in educational settings<br>• Need for rare disease events as social, educational, and networking opportunities for patients, families, HCP's, and researchers<br>• Need to explore multiple avenues of dissemination of research opportunities and communication.<br>• Lack of true accessibility in facilities deemed accessible<br>• Need for more awareness among HCPs
Theme 4: Current administrative systems are barriers to collaborative rare disease research.	• Ethical procedures require streamlining to facilitate research<br>• Hospital ethics procedures are particularly slow compared to institutions<br>• Northern Ireland is slower compared to other parts of the UK
Theme 5: Interprofessional collaboration was regarded as beneficial for rare diseases.	• Benefits of the multi-disciplinary approach discussed<br>• Need for cross-border collaboration<br>• International collaboration helpful for conditions were only a small number of people in one location are affected
